# Editorial: Early psychosis and early intervention: clinical, functional, and cognitive outcomes

**DOI:** 10.3389/fpsyt.2024.1471032

**Published:** 2024-08-26

**Authors:** Wing Chung Chang, Takahiro Nemoto, Sherry Kit Wa Chan, Charmaine Yu Zheng Tang, Young Chul Chung

**Affiliations:** ^1^ Department of Psychiatry, School of Clinical Medicine, Li Ka Shing Faculty of Medicine, University of Hong Kong, Hong Kong, Hong Kong SAR, China; ^2^ State Key Laboratory of Brain & Cognitive Sciences, University of Hong Kong, Hong Kong, Hong Kong SAR, China; ^3^ Department of Neuropsychiatry, Faculty of Medicine, Toho University, Tokyo, Japan; ^4^ Department of the Early Psychosis Intervention Programme (EPIP), the Institute of Mental Health Institute, Singapore, Singapore; ^5^ Department of Psychiatry, Jeonbuk National University, Medical School, Jeonju, Republic of Korea; ^6^ Research Institute of Clinical Medicine of Jeonbuk National University, Biomedical Research Institute of Jeonbuk National University Hospital, Jeonju, Republic of Korea

**Keywords:** early psychosis, early intervention, cognitive impairment, first-episode psychosis, clinical high-risk for psychosis (CHR), neurophysiologic abnormalities

Psychotic disorders are a group of severe mental disorders that affect 2%–3% of the population and constitute one of the leading causes of disability worldwide. Early intervention (EI) represents a major paradigm shift in psychiatric service and has been demonstrated to be effective in outcome improvement for first-episode psychosis (FEP) (1) and clinical high risk for psychosis [CHR-P, or termed at-risk mental state (ARMS)]. Nonetheless, substantial evidence has shown that a significant proportion of people with early psychosis still experience suboptimal clinical outcome, functional impairment, and cognitive dysfunction. This Research Topic, which comprises a series of articles specifically focusing on early psychosis, aims to explore and clarify the complex inter-relationships among symptomatology, psychosocial functioning, and cognitive deficits in the early course of psychotic disorders, so as to address potential research gaps and facilitate the development of more targeted interventions to further enhance treatment outcomes of this vulnerable population.

It is well-recognized that psychotic disorders are associated with cognitive impairment across multiple cognitive domains (2). Importantly, cognitive impairment is a major determinant of functional outcome. Several articles of this Research Topic specifically investigated cognitive functioning in FEP and CHR-P, and its relationship with clinical features and psychosocial functioning. Kim et al. evaluated the association between cognitive functioning and suicidal ideation in a cohort of recent-onset schizophrenia-spectrum disorders (SSDs). The study categorized patients with SSD into those with versus without suicidal ideation and compared these two groups with traditional risk factors, such as hopelessness, depressive symptoms, resilience levels, and perceived stress, and a comprehensive battery of cognitive functions. The results showed that patients with SSD who exhibited better cognitive abilities (especially executive functions, verbal and visual learning, and social cognition) were more prone to experiencing suicidal ideation, thereby highlighting the need to take into consideration cognitive functions in suicide risk evaluation, particularly those patients who have traditional risk factors and good cognitive functions. Alternatively, Mackinley et al. adopted a novel automated speech analysis coupled with Bayes network analysis in an antipsychotic-naïve FEP sample to explore the potential clinical utility of speech and communication deficits as targets for EI and functional outcome enhancement. Their results demonstrated that baseline speech production, but not other linguistic variables, significantly predicted NEET status (i.e., non-engaged in employment, education, or training) after 6–12 months of treatment commencement. This speech production measure was also indirectly related to global functional level. The findings suggest that impoverished speech, even at the subclinical level, may constitute important prognostic value for functional outcomes in early psychosis. Kam et al. aimed to disentangle cognitive heterogeneity in a group of adult patients with FEP by using data-driven cluster-analytic approach and identified three distinct cognitive clusters, namely, globally impaired (34.9%), intermediately impaired (38.8%), and relatively intact (26.3%) cognition subgroups, compared to demographically matched healthy controls’ performance. Importantly, these cognitive subgroups were differentially associated with demographic and illness-related variables. In particular, the globally impaired subgroup was older and displayed greater symptom severity, poorer insight, and worse subjective quality of life than the other two cognitive subgroups. Given the cross-sectional nature, future longitudinal research delineating patients into different cognitive trajectories and their relationships with clinical and functional outcomes would be particularly informative in treatment outcome prediction and development of tailor-made interventions to alleviate cognitive impairment in those at high risk for poorer cognitive functions in the early stage of illness. There is a paucity of research directly contrasting cognitive functions across established psychotic disorder and clinical and genetic high-risk (GHR) samples. Dong et al. presented a cross-sectional study comparing cognitive functions between first-episode schizophrenia (FES), CHR-P, and individuals at GHR for schizophrenia, relative to healthy controls. They found that FES, CHR-P, and GHR samples had significantly worse cognitive performance than controls in most of the cognitive domains. Notably, CHR-P and GHR showed no significant between-group difference across all cognitive domains, but demonstrated intermediate level of cognitive function in processing speed and attention/vigilance domains, relative to FES and controls, indicating that these two specific cognitive domains may represent cognitive markers indicating the risk for psychosis development. Of note, several issues in relation to cognitive impairment in FEP and CHR-P merit further discussion. First, a recent meta-analysis has indicated greater variability in cognitive functioning in individuals with FEP than in healthy participants, and suggested that subgroups of patients experience more severe disease-related cognitive dysfunction (3), which is in line with Kam et al. Second, although no differences in longitudinal cognitive changes between FEP and control groups were found, which suggests no evidence of continued cognitive decline (i.e., more akin to neurodevelopmental hypothesis rather than neuro-progressive hypothesis), this meta-analysis also revealed association between longer follow-up periods and greater cognitive decline in FEP samples. Given that the vast majority of the published data were based on studies with short follow-up durations, further research is required to clarify whether there is a subgroup of patients having a progressively deteriorating trend in cognitive functions along the course of illness, and if so, what are the potential risk factors or biomarkers for predicting declining cognitive trajectory, thereby facilitating early tailor-made cognitive remediation. Third, caution should be exercised in interpreting the findings of cross-sectional research on cognitive functions in CHR-P, which comprises a small proportion of at-risk individuals who will convert to full-blown psychosis as well as a majority of individuals who are non-converters. Hence, the profile and magnitude of cognitive impairment in CHR-P is indicative of both psychosis-specific vulnerability (based on converters) and transdiagnostic deficits (based on non-converters, comprising individuals with non-psychotic psychopathologies or even common mental disorders, and remitters from CHR-P) (4).

Identification of robust biomarkers in the early course of psychotic disorders will significantly enhance outcome prediction and disorder-subtype characterization. Ding et al. have examined a prepulse inhibition (PPI), a sensorimotor gating deficit, in the FEP sample by using a modified PPI paradigm, incorporating subjective attention component, and demonstrated enhanced discriminant validity for FEP relative to controls. The results also showed that perceived spatial separation PPI (termed PSS-PPI) was associated with symptoms and cognitive performance in patients with FEP, suggesting that PSS-PPI may be a useful biomarker for evaluating psychopathological symptoms in early psychosis. Arai et al. investigated exploratory eye movements (EEMs) and their relationships with white matter integrity, as measured by fractional anisotropy (FA) of superior thalamic radiation (STR; which connects frontal eye fields and thalamus) by diffusion tensor imaging (DTI) in individuals with attenuated psychosis syndrome (APS, a subgroup of CHR-P). Individuals with APS exhibited aberration EEMs relative to healthy controls, and EEM parameters including mean and total eye scanning length were related to STR alterations, thereby underscoring the idea that impairment of STR may contribute to the neurobiological mechanisms underlying manifestations of CHR-P and its related oculomotor disturbances. Further research with a larger sample size and using a prospective design will clarify the potential value of EEM in predicting psychosis and functional outcome. Aeberli et al. examined deficits in mismatch negativity (MMN) across various at-risk subgroups encompassing individuals with CHR-P, individuals with basic symptoms (BS) only, and individuals fulfilling both CHR-P and BS criteria. This study revealed that all three risk groups showed significantly lower MMN activity at frontal source compared with healthy controls. Further analysis suggested that this specific deficit was significantly associated with psychosis transition at the 3-year follow-up (albeit based on a small sample of 15 participants who converted to psychosis). In sum, the results indicate that MMN deficit occurs already early in the course of the disease, as indicated by its presence in the BS risk group, and frontal MMN changes may be particularly relevant for predicting psychosis transition in at-risk groups. Although these studies indicate the potential utility of neurophysiological deficits as disease biomarkers for psychotic disorders, including psychosis prediction, it should be noted that few candidate predictors reached a level of evidence sufficient to inform clinical practice regarding prediction of CHR-P to full-blown psychotic disorders (5). Different neurophysiological measures may also be differentially associated with the nature (e.g., clinical versus genetic risk marker) and the degree of psychosis risk (6), and a combination of neurophysiological measures would likely yield an enhanced prediction model. Moreover, progression from at-risk status to psychosis is a dynamic developmental process, involving complex longitudinal interplays between multiple variables and risk factors. In this regard, earlier static models with candidate predictors derived on the basis of a single time point (baseline assessment) will unlikely generate a clinically applicable and accurate prediction algorithm. Application of dynamical prediction modeling, taking into consideration longitudinal, multiple time-point measurements, would improve psychosis and outcome prediction (7).

Substantial evidence has shown that psychotic disorders are associated with markedly elevated risk of premature mortality, physical comorbidity, and shortened life expectancy, compared with the general population (8, 9) Chua et al. measured weight trajectory patterns among patients who received FEP service over the first 2 years of treatment and demonstrated that a majority of patients belonged to the high-risk groups for clinically significant weight gain (38.6% as super high risk; 34% as high risk mitigated). The results highlight the importance of adopting early, preemptive strategies in the initial phase of treatment commencement for FEP to promote physical health and ensure adherence to guideline-concordant monitoring of cardiometabolic parameters on a regular basis to facilitate early detection and prompt interventions for those at high risk for obesity and metabolic syndrome. Maechling et al. conducted a systematic review on mobile health strategies for the management of FEP. The review is timely as mobile or digital health intervention has increasingly been applied in mental disorders, including early psychosis (10), and has the potential to further enhance the quality of and engagement with EI service for young people with FEP. Overall, the review affirmed the preliminary efficacy of various types of mobile health applications, including symptom monitoring, enhanced service engagement, and promoting the self-management of the illness and the recovery phase of FEP. However, major limitations are noted including the lack of randomized controlled trials and a small sample size. Moreover, ethical issues regarding data protection and patient privacy, as well as lack of consensus or regulations regarding mobile health applications, warranted further exploration and discussion.
